# Characterization of the complete mitochondrial genome of short-tailed field vole, *Microtus agrestis*

**DOI:** 10.1080/23802359.2018.1467240

**Published:** 2018-08-13

**Authors:** Jin-Qing Jiang, Shi-Xiu Wu, Jun-Jie Chen, Chang-Zhong Liu

**Affiliations:** College of Animal Science and Veterinary Medicine, Henan Institute of Science and Technology, Xinxiang, China

**Keywords:** Short-tailed field vole, *Microtus agrestis*, mitochondrial genome, phylogenetic analysis

## Abstract

In this study, the complete mitochondrial genome of short-tailed field vole, *Microtus agrestis,* was determined through sequencing of PCR fragments. The complete mitochondrial genome of *M. agrestis* was 16,538 bp in length and encoded 13 protein-coding genes, 22 transfer RNA (tRNA) genes, and two ribosomal RNA genes. The overall nucleotide composition is: 15.7% A, 27.5% T, 25.5% C, and 31.4% G, with a total G + C content of 56.9%. By phylogenetic analysis using Bayesian method, *M. agrestisa* showed the closest relationship with the southern vole (*Microtus rossiaemeridionalis*).

The short-tailed vole (*Microtus agrestis*) is a grey-brown vole, around four inches (10 cm) in length, with a short tail. It is one of the most common mammals in fields, with a range extending throughout Western Europe and eastwards to Lake Baikal in Siberia and north west China and northward to Norway, Sweden and Finland (Wilson et al. 2005).

In this study, the specimen was collected from the northern Junggar basin (86.78 E, 48.69 N), Xinjiang Province, China. The total DNA was extracted from muscular tissue, using the commercial Animal Tissues Genomic DNA Extraction Kit (Solarbio, BJ, CN) following the manufacturer’s instructions, and then used as the template for polymerase chain reaction (PCR) amplifications. The isolated DNA was stored in the sequencing company (BGI Tech, Shenzhen, China). The amplified products were sequenced using the amplification primers. All sequencing was done by a commercial sequencing service (BGI Tech, Shenzhen, China). Some small number of PCR products that could process complex secondary structures or high A + T content, were cloned into the PMD-19T vector (TaKaRa, Dalian), then transformed to JM109 competent cell (TaKaRa, Dalian) for sequencing. All sequencing sequences were assembled with the program Seqman in the DNASTAR package (Burland 1999).

Protein coding genes were predicted using the MITOS web server (Bernt et al. 2013) using the vertebrate mitochondrial genetic code. Protein-coding regions and ribosomal RNA genes were also annotated manually and confirmed by comparison to the mitogenome of *Microtus ochrogaster* (Genbank accession: NC_027945) that available in Genbank. The transfer RNA (tRNA) genes were identified and assigned putative secondary structures using the program tRNAscan-SE (Lowe et al. 1997) or by manually identifying potential secondary structures and anticodon sequences through visual inspection. The graphical map of the complete mitochondrial genome was drawn using the online sofware OrganellarGenomeDRAW (Lohse et al. 2007).

The complete mitogenome of *Microtus agrestis* (Genbank accession: MH152570) is a closed-circular molecule of 16,538 bp in length, which is a litter longer than other sequenced *Microtus* mitogenomes. It presents the typical set of 37 genes observed in metazoan mitogenomes, including 13 PCGs (*cox*1-3, *co*b, nad1-6, *nad*4L, *atp*6, and *atp*8), 22 tRNA genes (one for each amino acid, two each for Leucine and Serine), two genes for ribosomal RNA subunits (*rrn*S and *rrn*L). The gene arrangement of this mitochondrial genome was the same as that of other *Microtus* species. Genome organization of *M. agrestis* is very compact, encoding 15,489 bp functional regions (including D-loop region).

For phylogenetic analysis assessing the relationship of this mitogenome, we selected other 31 related Muroidea mitogenomes downloaded from GenBank. The genome-wide alignment of all mt genomes was done by HomBlocks (Bi et al. 2018), resulting in 10,714 positions in total, including almost all whole or partial PCGs and rRNA genes. The whole genome alignment was analyzed by PhyloBayes ver. 3.3 (Lartillot et al. 2012) under the CAT-GTR + Γ model. Two independent MCMC analyses were run for 10,000 cycles in PhyloBayes. Convergence was checked based on time-series plots of the likelihood scores using Tracer (http://tree.bio.ed.ac.uk/software/tracer/). The first 5000 cycles were discarded as burn-in, and the remaining trees were summarized to obtain Bayesian posterior probabilities (BPPs). The resulting tree was represented and edited using FigTree v1.4.1 (http://www.umiacs.umd.edu/∼morariu/figtree/). As shown in Figure 1, the phylogenetic positions of these 32 mt genomes were successfully resolved with full BPPs supports across almost all nodes. As expected, *M. agrestis* showed the closest relationship with the southern vole (*Microtus rossiaemeridionalis*).

**Figure 1.**
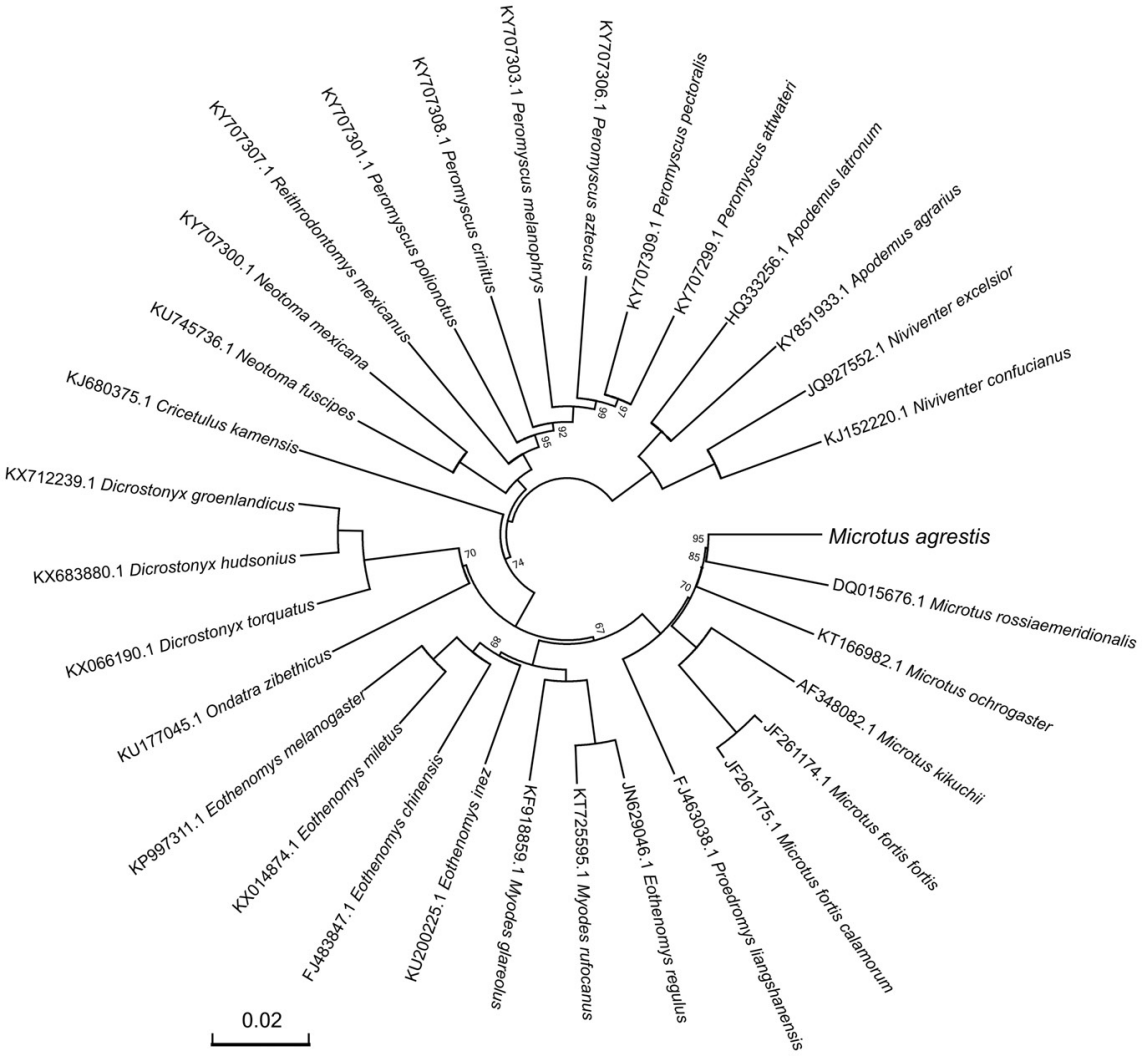
Phylogenetic tree yielded by Bayesian analysis of 32 Muroidea mitochondrial genomes. This tree was drawn without setting of an outgroup. Nodes exhibit 100% posterior probability (PP) were not shown. The length of branch represents the divergence distance.
